# Supplementary figures and images for: A role for gorilla APOBEC3G in shaping lentivirus evolution including transmission to humans
Source: PLoS Pathog. 2020 Sep 10;16(9):e1008812. doi: 10.1371/journal.ppat.1008812 (PMC7482973; doi:10.1371/journal.ppat.1008812)

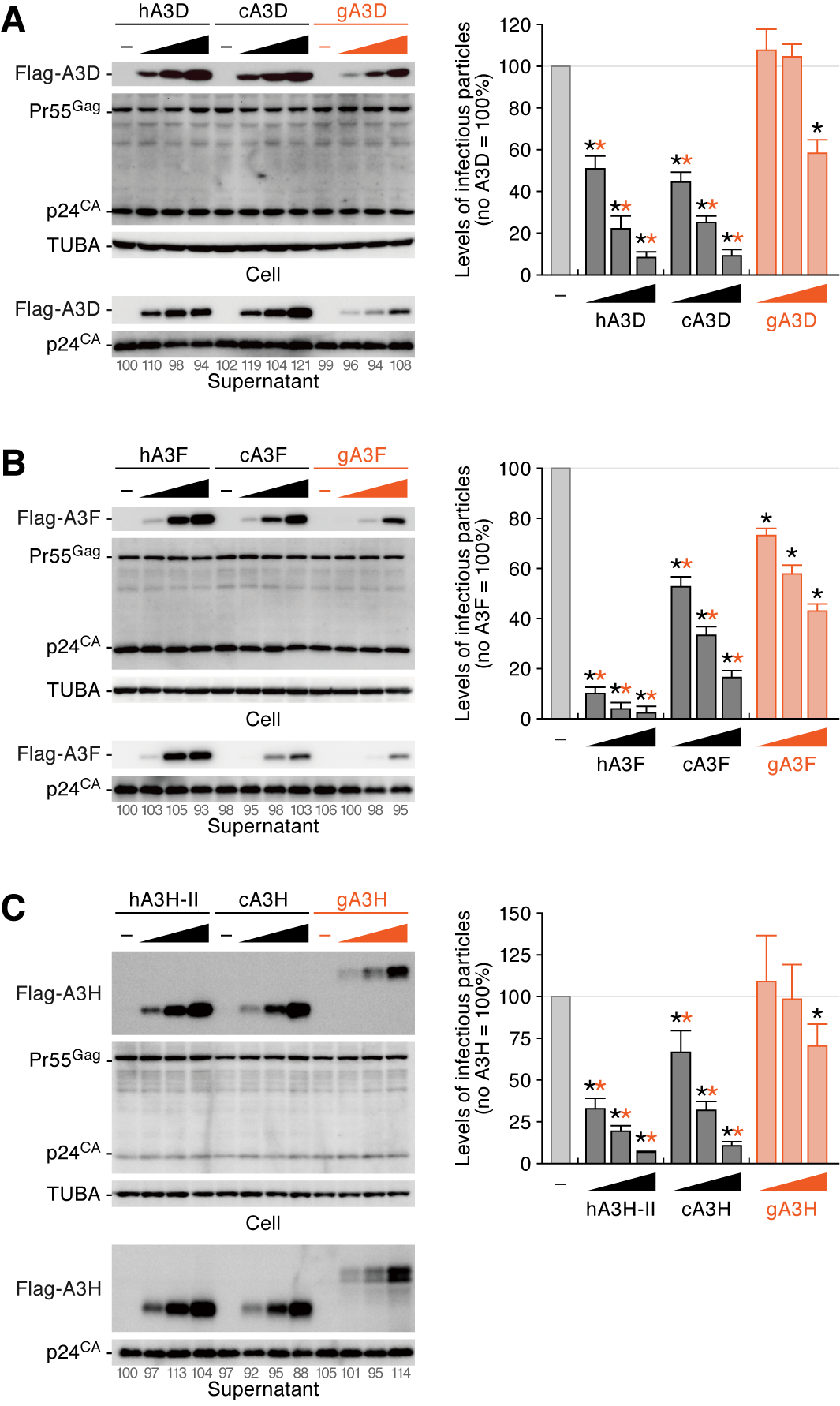

Supplement: S1 Fig — HEK293T cells were co-transfected with pNL4-3Δvif (500 ng) and the different amounts of expression plasmids for great ape A3D (A), A3F (B) and A3H (C) (0, 50, 100, and 200 ng; the plasmid amount was normalized by empty vector). Cells and supernatants were harvested at two days post-transfection and were used for Western blotting (left) and TZM-bl assay (right). For Western blotting, the band intensity of viral p24 was quantified and the intensity value of the leftmost lane was set to 100%. For TZM-bl assay, the infectivity value without A3 was set to 100%. (TIF) [file ppat.1008812.s001.tif]

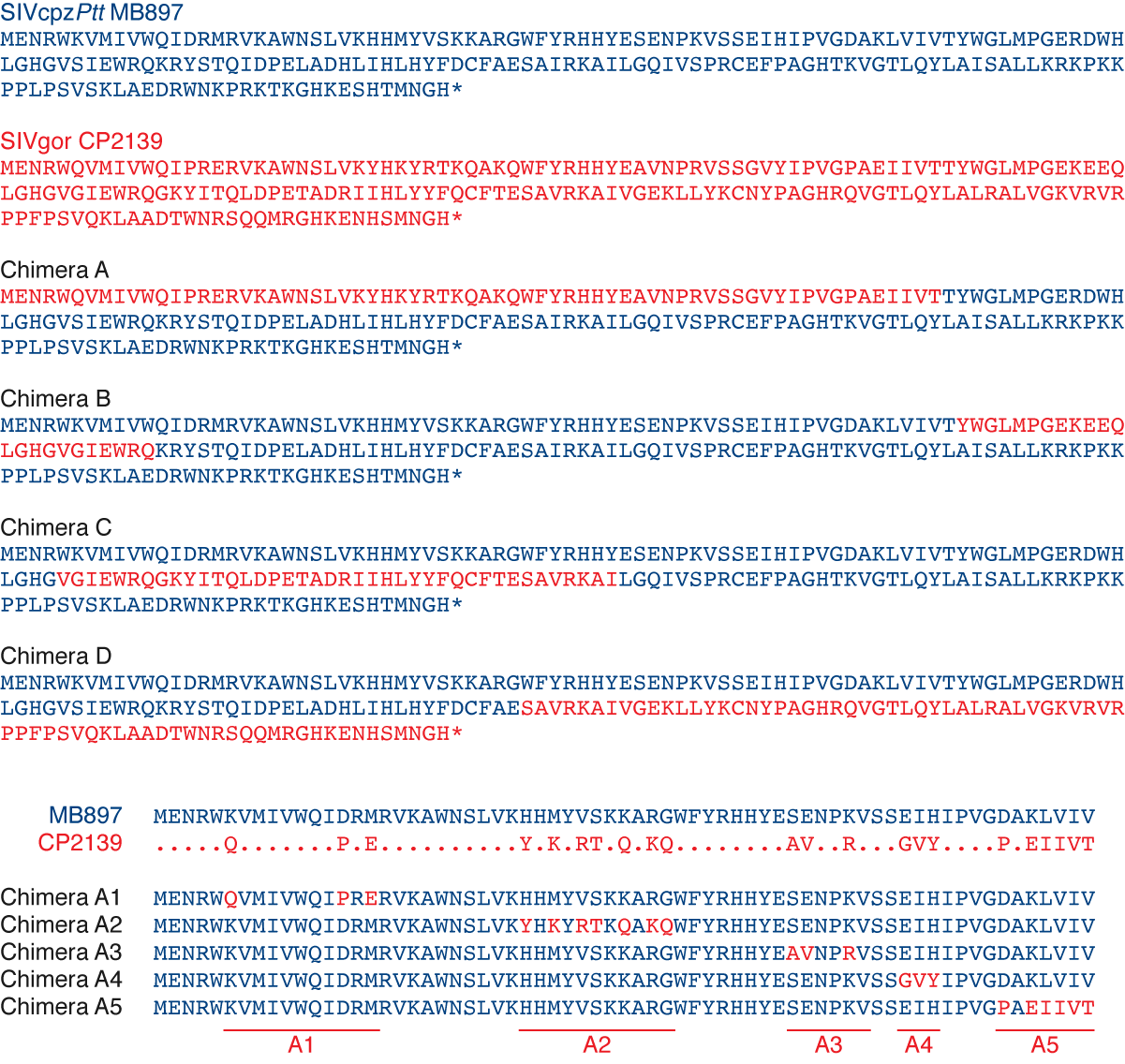

Supplement: S2 Fig — The scheme of respective mutants is also shown in Fig 2A. (TIF) [file ppat.1008812.s002.tif]

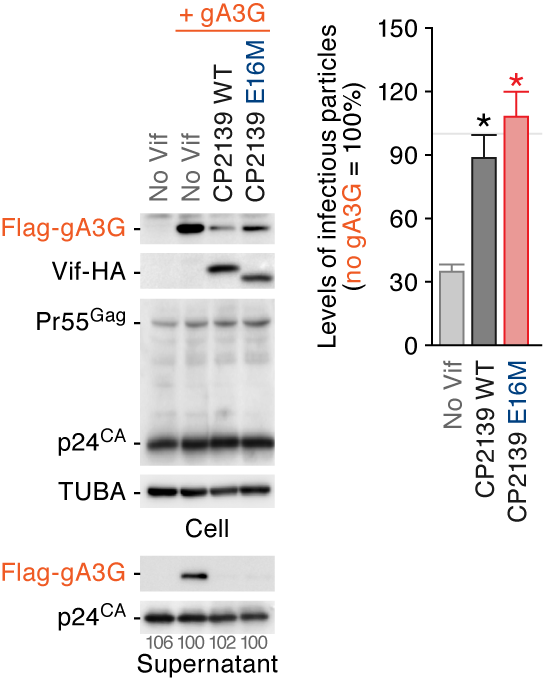

Supplement: S3 Fig — HEK293T cells were co-transfected with pNL4-3Δvif (500 ng) and the expression plasmids for gA3G (50 ng) and indicated Vif (500 ng). Cells and supernatants were harvested at two days post-transfection and were used for Western blotting (left) and TZM-bl assay (right). For Western blotting, the input of cell lysate was standardized to TUBA, and representative results are shown. The band intensity of viral p24 was quantified and the intensity value of the gA3G expressing cells without Vif (second from the left) was set to 100%. For TZM-bl assay, the percentage of the value without gA3G is shown. The mean values of three independent experiments ± SEM are shown. (TIF) [file ppat.1008812.s003.tif]

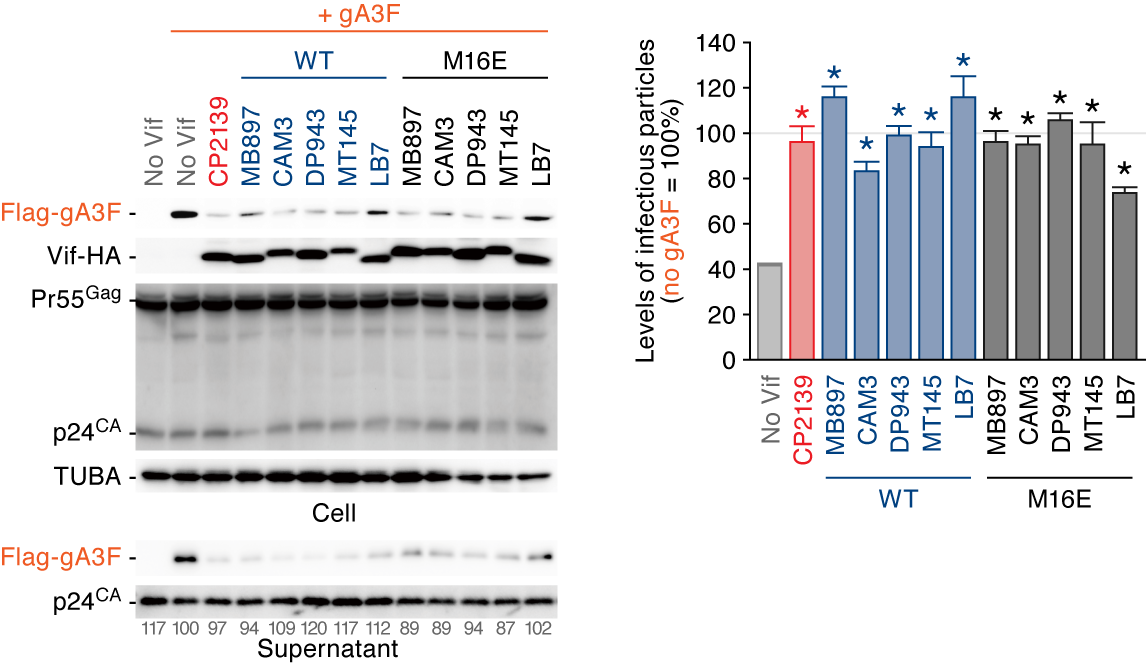

Supplement: S4 Fig — HEK293T cells were co-transfected with pNL4-3Δvif (500 ng) and the expression plasmids for gA3F (200 ng) and indicated Vif (500 ng). Cells and supernatants were harvested at two days post-transfection and were used for Western blotting (left) and TZM-bl assay (right). For Western blotting, the input of cell lysate was standardized to TUBA, and representative results are shown. The band intensity of viral p24 was quantified and the intensity value of the gA3F expressing cells without Vif (second from the left) was set to 100%. For TZM-bl assay, the percentage of the value without gA3F is shown. The mean values of three independent experiments ± SEM are shown, and statistically significant differences (P < 0.05) versus "no Vif" are shown by asterisks. (TIF) [file ppat.1008812.s004.tif]

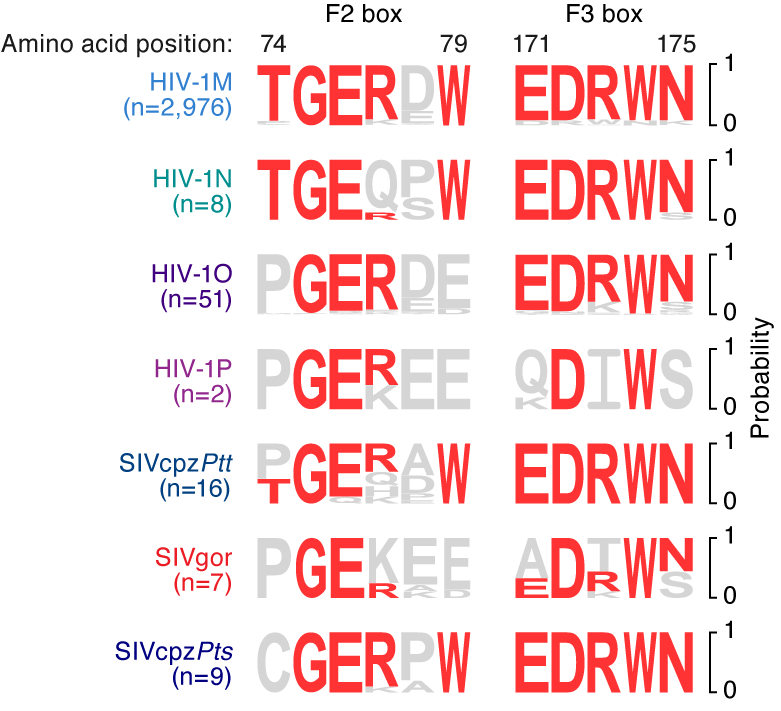

Supplement: S5 Fig — Logo plots of the Vif residues between 74–79 (corresponding to the F2 box in HIV-1M; left) and the residues between 171–175 (corresponding to the F3 box in HIV-1M; right) in HIV-1MNOP, SIVcpz and SIVgor are shown. The number in parenthesis (n) indicates the number of viral sequences used in this analysis. (TIF) [file ppat.1008812.s005.tif]
