# Supplementary material for: A role for gorilla APOBEC3G in shaping lentivirus evolution including transmission to humans
Source: PLoS Pathog. 2020 Sep 10;16(9):e1008812. doi: 10.1371/journal.ppat.1008812 (PMC7482973; doi:10.1371/journal.ppat.1008812)
Supplement: Attachment — Submitted filename: 200714 Reply to PP reviewers 6 (RC).pdf [file ppat.1008812.s012.pdf]

Review Commons

Reviewer #1 (Evidence, reproducibility and clarity (Required)):

Nakano and colleagues study how APOBEC3G may be responsible for the restriction of transmission of SIV from chimpanzees (cpz) to gorillas (gor). Using transfection of 293T cells with HIV-1 IMCs, Western Blot and TZM-Bl infectivity assays, they show that SIVcpzPtt Vif weakly counteracts gorilla A3G (gA3G), whereas SIVgor Vif does counteract gA3G, as expected. They tested only one strain of SIVcpzPtt. Using chimeras of SIVcpz and SIVgor Vif, and mutants of SIVcpz Vif, they show that mutating the methionine at position 16 to a glutamic acid restores the ability of SIVcpz Vif to counteract gA3G. They confirm this observation with Vif from other SIVcpzPtt strains. Using modelisation from known HIV-1M Vif structure, they give structural insights into how this residue 16 might impact the surrounding residues and modify Vif surface. In the last part, they confirm the importance of the YRHHY motif to the counteraction of A3G. They show that the DRMR conserved motif, important for A3F counteraction, is lost in SIVgor Vif (and HIV-1 O and P derivatives), although it is still able to counteract A3F. Thus, gA3F is degraded by SIVgor Vif through a mechanism that is independent of this motif. This last part does not add anything substantial to the overall message of the manuscript concerning transmission of HIV from chimpanzee to gorilla. This would require more mechanistic insights into how the M16E mutation modifies the mode of action of SIVgor Vif. Overall, results are convincing and well-presented, the paper is well-written and the results bring interesting insights into SIV transmission from chimpanzee to gorilla.

Major comments:

1. Are TZMbl assays normalized for p24 inputs? If not, it would be better to use terms like "levels of infectious particles" because infectivity per se is not measured.

Our reply

According to the reviewer’s suggestion, we relabeled the y-axes of the TZMbl assay data in the revised manuscript. Regarding the amount of p24 input; as shown in all of the immunoblotting, the levels of p24 in culture supernatants were constant (ie, the expressions of Vif and APOBEC3 did not affect the level of released viral particles, consistent with previous reports).

Viral p24 immunoblot signals are already shown in the figures of revised manuscript. Furthermore, we have now used p24 ELISA to further quantify p24 levels in culture supernatants from the experiments shown in **Fig 4F-4H**. As shown in **Fig R1** below, these ELISA results show that the amounts of released virions were comparable. These results further indicate that the differences in viral infectivity shown in **Fig 4F-4H** are not due to the decrease of viral p24 by the expressions of either Vif or APOBEC3 but are due to the APOBEC3F proteins incorporated into the virions.

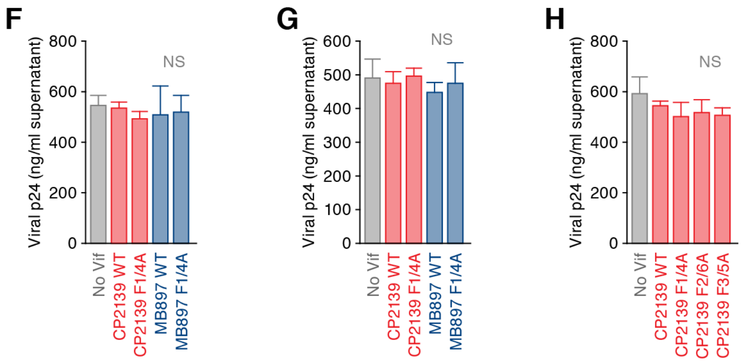

Fig R1. The amounts of viral p24 in the culture supernatants harvested at two days post-transfection were measured by p24 ELISA kit (ZeptoMetrix). These three panels correspond to those shown in **Fig 4F-4H** of revised manuscript. The mean values of three independent experiments ± SEM are shown, and statistically significant differences versus "no Vif" are analyzed by Student's t test. NS, no statistical significance.

2. Figure 1, inclusion of a broader panel of SIVcpzPtt Vif would allow to conclude that "gA3G could not be neutralized efficiently by SIVcpzPtt Vif". Moreover, including SIVcpzPtt Vif that are closer to SIVgor Vif (for example MT145) would be informative. A control showing that SIVcpzPtt Vif is functional against cA3G would also be useful.

Our reply

Regarding *"inclusion of a broader panel of SIVcpzPtt Vif would allow to conclude that "gA3G could not be neutralized efficiently by SIVcpzPtt Vif"*; in previous studies, similar data were already shown. For instance, D'arc et al. showed that a variety of SIVcpzPtt Vif (strains EK505, LB715, MT143, Gab2 as wells as SIVcpzPts Vif (strains Tan1, Tan2, Tan3) can neutralize gorilla A3G (D'arc et al, PNAS, 2015 PMID 25733890; Letko et al, JVI, 2013 PMID 23986590). We have acknowledged these prior reports in our revised manuscript (page 6, lines 133-134).

Regarding *“including SIVcpzPtt Vif that are closer to SIVgor Vif (for example MT145) would be informative”*; to satisfy the Reviewer 3’s request, we re-aligned the sequences and re-constructed the ML phylogenetic tree. As shown in the Fig. 1A of revised manuscript, MT145 was an outgroup of SIVcpzPtt and SIVgor but was not close to SIVgor Vif. These data suggest that MT145 is not related to the cross-species lentiviral transmission events in great apes.

Regarding *“A control showing that SIVcpzPtt Vif is functional against cA3G would also be useful”*; related to the initial comment, previous studies (D'arc et al, PNAS, 2015 PMID 25733890; Letko et al, JVI, 2013 PMID 23986590) have already addressed this issue.

3. Figure 2, does reversion of SIVgor Vif E16 to an M render It unable to counteract gA3G?

Our reply

To satisfy the reviewer’s concern, we performed this experiment using the E16M mutant of SIVgor CP2139 Vif. These results are shown below (**Fig R2**) as well as in **S3 Fig** of the revised manuscript:

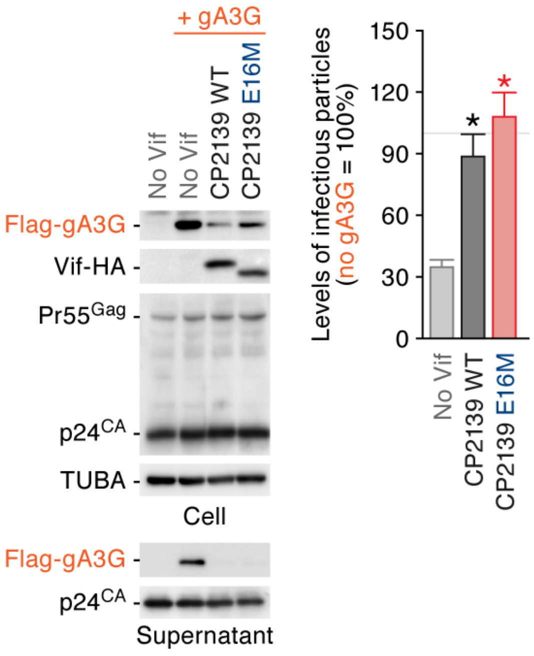

Fig R2. HEK293T cells were co-transfected with pNL4-3Δvif (500 ng) and the expression plasmids for gA3G (200 ng) and indicated Vif (500 ng). Cells and supernatants were harvested at two days post-transfection and were used for Western blotting and TZM-bl assay. For Western blotting, the input of cell lysate was standardized to TUBA, and representative results are shown. For TZM-bl assay, the percentage of the value without gA3G is shown. The mean values of nine independent experiments ± SEM are shown, and statistically significant differences ($P < 0.05$) versus "no Vif" are shown by asterisks. A gray horizontal bar indicates 100%.

As shown above, the ability of the E16M mutant to degrade gA3G seems to be

lower than that of wild-type Vif. However, the level of gA3G in virions and viral infectivity of the E16M mutant were comparable to those of wild-type Vif. We are not sure about the reason of the discrepancy on the levels of gA3G between cells and virions, but similar results were reported in previous studies (Yu et al., J Biol Chem, 2004 PMID 15466872; Schröfelbauer et al, PNAS, 2004 PMID 14978281; Gaur and Strebel, PLOS One, 2012 PMID 23189135), and the result is pasted below:

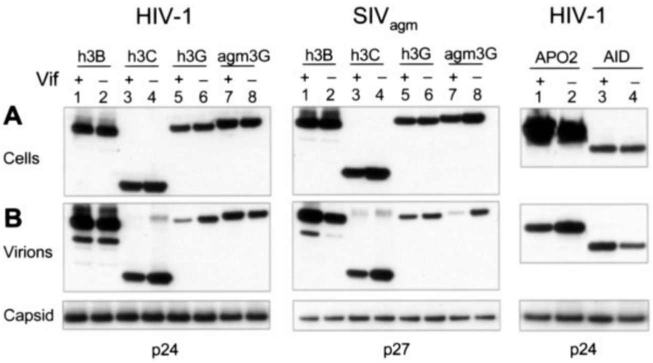

FIG. 1. **APOBEC3B and APOBEC3C are encapsidated into HIV-1 and SIV$_{agm}$ virions.** Virions were generated by cotransfection of 293T cells with wild-type or Δ*vif* HIV-1 or SIV$_{agm}$ DNA and HA-tagged APOBEC expression vector. Human APOBEC3B, APOBEC3C, APOBEC3G, AGM APOBEC3G, and APOBEC2 are labeled *h3B*, *h3C*, *h3G*, *agm3G*, and *APO2*, respectively, in this and subsequent figures. *A*, APOBEC expression in the transfected cells was detected on an immunoblot probed with anti-HA mAb. *B*, encapsidated APOBEC in virions normalized by p24/p27 enzyme-linked immunosorbent assay was detected on a parallel immunoblot, labeled as in *panel A*. The presence or absence of Vif in the viruses is indicated as + and −, respectively. Equal loading of virions on the immunoblots was confirmed with antibody to the HIV and SIV capsid.

In the middle panel, the level of agmA3G in the SIVagm Vif-cotransfected cells (lane 7) is comparable to that in mock-cotransfected cells (lane 8). However, the level of agmA3G in the virions released from the SIVagm Vif-cotransfected cells (lane 7) is clearly lower than that in mock-cotransfected cells (lane 8). Collectively, there may be factor(s) that is associated with the efficacy of A3 incorporation into the released virions, which is independent of the Vif's activity to degrade A3 proteins in the cells. However, pursuing and characterizing such factor(s) would be beyond the scope of our current study.

These data also indicate that the E16M mutation of SIVgor Vif does not revert its ability to degrade gA3G, and further suggest that SIVgor Vif has acquired additional mutations, which can fortify and/or compensate the ability of SIVgor Vif to degrade gA3G, during the adaptation to gorilla. Nevertheless, our results shown in **Fig 2** of revised manuscript strongly suggest that the M16E mutation confers the ability to degrade gA3G on SIVcpz Vif. We are confident in the conclusion that the acquisition of M16E mutation in Vif is a major step for SIVcpz to adapt to gorilla. Moreover, based on our additional data above, we discussed this issue in the revised manuscript (page 10, lines 225-231; pages 14-15, lines 334-345), according to the suggestion from **Reviewer 2 of PLOS**

Pathogens.

4. Figure 3A, SIVgor BQID2 Vif has a Q at position 16, not an E. Is it able to counteract gA3G?

Our reply

Please see the *vif* sequence of SIVgor strain BQID2 (accession no. KP004991):
atggaaaacaaatggcaggt**R**ctgatagtatggcaag**W**ggacagacagaaggtgaaagcatggaaca
gcctggtgaaataccataagtataggtccaaaaaggccaaagattggttttacagacaccattatgaagccac
aaatccaagagtcagttcaggtgtatatattccagtaggtggtgcttgcataatagtgaccacatattggggtttg
atgcca**R**gggaag**M**agctga**R**caattgggaaatggggttagtatagaatggcagctaaggaattatagaa
cacagattgatcctgaaacagcagacaggatgatacacctgtattat**Y**tttc**M**tgttttacagagtcagcaatc
aggaaagccatcttagggcaaaaggtgttacccacttgtgaataccctgcaggacatagtcaggtagggacc
cttcaattcctagctttgagggtattagtaaaaacaagaaaatttagacctcccttacccagtgtccagaagttag
cagaagatagatggaacaggtacccgaggatcagggaccagctagagagccattcaatgaatgggcactg
gaagttctggaagaactga

As shown above, 7 out of the 597 ORF nucleotides were ambiguous (e.g., R, W, M or Y), and therefore, 4 out of the 7 codons containing these ambiguous nucleotides were unable to be defined as the codon encoding single amino acid (for instance, there are 2 "X"s in the amino acid sequence of BQID2 in Fig. 2I of the revised manuscript [Fig. 3A of the previous version]. We noted that "X is an undefined amino acid because of nucleotide ambiguity" in the legend of revised manuscript [page 34, line 783]).

We also found that the full-length sequence of SIVgor strain BQID2 (9,241 nucleotides; accession no. KP004991) contained 53 ambiguous nucleotides. These observations suggest that the sequence quality of this strain is not so good, and therefore, a Q at position 16 of BQID2 Vif may also due to the low quality of sequence. The sequence ambiguity may affect the experimental result, and it was difficult for us to choose an "authentic" BQID2 sequence from the public database for artificial DNA synthesis and subsequent virological experiments. Therefore, we could not address this issue. We explained this possibility in the revised manuscript (page 9, lines 210-215). We think the editor and reviewers for understanding this explanation.

5. The models presented in Figure 3 does not address Vif-A3G interactions. Are there available structures of SIV Vif and great ape A3G that could be used to infer how modification at position 16 may impact the interaction?

Our reply

We appreciate the reviewer's comment. However, the crystal structures of both of SIV Vif and gorilla A3G are not solved yet. Therefore, to address the interface of SIV Vif and gorilla A3G, both structures would require homology models. The reliability of the docking model of two homology models would be generally low, and the result would be too speculative. We hope to address this issue with future studies and thank the editor (and reviewers) for understanding this situation.

Minor comments:

1. In Figure 1C, cutting the axis would make the dose response clearer. For that panel, how many experiments were performed? Is it possible to perform any statistics?

Our reply

According to the reviewer's suggestion, we modified the Figure in the revised manuscript. We noted that gA3G is less antiviral than hA3G and cA3G in the revised manuscript (page 7, lines 157-158). We also applied statistical analyses on these data.

Regarding *"how many experiments were performed?"*; these are the result of three independent experiments. We also applied statistical analysis. We clarified these issues in the revised manuscript (pages 32-33, lines 753-757).

2. Results presented in Figure 3B could be included in Figure 2 to confirm the results obtained with one strain of SIVcpzPtt.

Our reply

According to the reviewer's suggestion, the indicated figure was included in **Fig 2** of the revised manuscript. Related to this, **Fig 3A** of the previous version was

also included in **Fig 2** of the revised manuscript.

Reviewer #1 (Significance (Required)):

cf above

Review Commons
Reviewer #2 (Evidence, reproducibility and clarity (Required)):

Summary:

The authors investigate the ability of SIVcpz to transmit to SIVgor. They find that gA3G can restrict SIVcpz, thus providing a cross species barrier that was overcome. This could be overcome by having an M16E mutation in SIV Vif. Sequence alignment shows the conserved M16 in SIVcpz Vif changed to E16 in SIVgor Vif. The authors also find that SIVgor Vif induces degradation of A3F in a DRMR independent manner.

Major comments:

The key conclusions are supported by the data. However, the DRMR independent degradation could be investigated further. The DRMR Vif of HIV-1 may participate in A3F degradation indirectly (Richards et al., Cell Reports, 2015). Thus, there may be other compensatory interactions in the relavent region of Vif that maintain the electrostatic interactions. Other mutations in SIVgor Vif would be helpful to determine what are essential interacting residues.

Our reply

First of all, we would like to appreciate the reviewer for considering that the key conclusions are supported by the data.

To satisfy the reviewer's concern (*"Other mutations in SIVgor Vif would be helpful to determine what are essential interacting residues"*), we performed additional three experiments. First, we showed that the M16E Vif mutants of the 5 strains of SIVcpz*Ptt* including MB897 were able to degrade gA3F (**S4 Fig** of the revised manuscript). This additional result suggests that the M16E substitution does not affect the Vif's ability to degrade gA3F. Second, to assess the possibility that the residues 14-17 in HIV-1O, HIV-1P and SIVgor can be compensatory for the DRMR motif (F1 box) of HIV-1M, we constructed chimeric mutants based on HIV-1M strain JRCSF Vif, which possess the amino acid residues derived from HIV-1O, HIV-1P and SIVgor: DRQK (from HIV-1O), SREK (from HIV-1P), PREK (from HIV-1P) and PRER (from SIVgor) (**Fig 4D** of the revised manuscript). Although parental HIV-1 JRCSF Vif counteracted hA3F, these chimeric mutants

did not (**Fig 4E** of revised manuscript). Together with the other findings, our data suggest that the importance of the F1 box is dependent on the species of A3F. Finally, we prepared two additional SIVgor Vif mutants, the F2/6A and F3/5A mutants, and revealed that the F2 box of SIVgor Vif plays a pivotal role in neutralizing gorilla A3F (**Fig 4H** of revised manuscript).

Moreover, we added a paragraph (regarding A3F) in the discussion section of revised manuscript (page 16, lines 376-382). We hope these additional results on the Vif's ability to neutralize A3F satisfy the reviewer's concern.

Reviewer #2 (Significance (Required)):

The work done is not novel in technique, but is novel in that the transmission event between SIVcpz and SIVgor had not been previously studied. The advance identifies a new amino acid in Vif that represents a cross-species barrier. The advance is conceptual; that A3Ggor responsible for the cross species barrier and that SIVgor Vif uses a different amino acid motif to induce degradation of A3F. The A3F portion could be the more significant part, but it is not a complete story. Also, whole manuscript is about A3G and then this small part of A3F at end; seems like two unfinished stories. Try to make the two story lines more cohesive.

Our reply

We would like to thank the reviewer for considering the novelty of our study. To satisfy the latter part of the comment, we tried to make the two story lines more cohesive. For that, we performed additional experiments according to the reviewer's comments (please see above). Moreover, we revised the manuscript to address the issues raised by the other two reviewers. We hope the revised manuscript is completed.

REFEREES CROSS COMMENTING

The reviews are fair and justified. I have no comments, as I am in agreement with other reviewers

Review Commons
Reviewer #3 (Evidence, reproducibility and clarity (Required)):

In this study, Nakano and colleagues bring novel findings on the adaptation of SIV from chimpanzee to gorilla, giving rise to SIVgor, which was itself at the origin of HIV-1 O and P in humans. In particular, they investigated how the viral antagonist Vif adapted to antagonize the gorilla restriction factor APOBEC3G (and APOBEC3F to a lesser extent).
They used an elegant approach with functional assays of diverse evolutionary-relevant Vifs/APOBEC3s, the use of many chimeras/mutants of lentiviral Vifs in the context of lentivirus replication, and structure homology modelling.
Their major finding is that they identified the amino acid mutation that confer SIVcpz Vif with the ability to restrict gorilla APOBEC3G (A3G).
A3G has been of particular interest as a restriction factor and species barrier against HIV/lentiviruses. Previous reports showed that gorilla A3G was the only known primate orthologue encoding a glutamine at position 129, in the Vif-interacting region, which constituted a barrier against SIV from other primates, and notably from chimpanzees (Letko et al 2013, D'arc et al 2015). Here, the authors performed extensive experiments using SIVcpz/SIVgor Vif chimeras and single point mutants to identify the M16E mutation as sufficient to confer SIVcpz Vif with the ability to degrade gorilla A3G. They further characterized the importance of this position and surrounding residues in terms of physicochemical properties and conformation. The experiments with vif chimeras and mutants are well constructed and results are very convincing.
The authors expanded their work to APOBEC3F antagonism and bring some new insights on SIVgor Vif - gA3F determinants.

Major comments:

While this study is very complete and convincing, it fails to correctly acknowledge the importance of previous studies and the authors should therefore significantly modify the Introduction, the first paragraph of the Results, and the Discussion to reflect what has been previously described and what this study brings to the field. Indeed, in 2013, Letko and colleagues already established gorilla A3G as a barrier against SIVcpz and other lentiviruses (Letko et al. JVI 2013; Figures 7-8). These findings were further examined in D'arc et al with a large panel of Vifs from

SIVcpz strains (D'arc et al. PNAS 2015). As such, most of Figure 1 and the first paragraph are confirmation of previous findings (most could be moved to Suppl Information), which should be made clearer throughout the manuscript.

In the same vein, a substantial effort should be made to better reference and acknowledge previous studies of major relevance to the subject, such as Letko et al. JVI 2013 and D'arc et al. PNAS 2015 on gorilla A3G, Etienne et al. Cell HM 2013 on the adaptation to great ape A3G, Richards et al Cell Rep 2015 and Letko et al Cell Rep 2015 on Vif-A3 interface, and update/revise references on the background of SAMHD1/Vpr/Vpx, A3F/lentivirus interface.

Our reply

First of all, we would like to appreciate the reviewer for considering that our study is very complete and convincing. At the same time, we are sorry for insufficient acknowledgment for previous studies. According to the reviewer's suggestion, we revised the manuscript. However, regarding *"most of Figure 1 and the first paragraph are confirmation of previous findings (most could be moved to Suppl Information)"*; we understand some part of the data shown in **Fig 1** are redundant to previous findings. However, we feel that showing these data in **Fig 1** is friendlier for readers to understand the story of our study. According to the reviewer's suggestion, we referred important previous studies in the revised manuscript to the best of our abilities. We hope the current version of the manuscript satisfies the reviewer's concern.

1. Concerning phylogenetics, the Methods and the Legends should be better defined. The model used for the construction of the vif phylogeny, as well as the number of bootstrap replications performed to assess statistical support of the branches, should be mentioned in the corresponding method section. Similarly, the method to calculate the "nt sequence similarity" and the "p-distance" should be given.

Our reply

We are sorry for the insufficient explanation. We modified the Methods and figure legends. Also, the method to calculate "p-distance" was included in the revised manuscript (page 19, lines 425-430). Because the meanings of "nt sequence similarity" and "p-distance" are the same, we omitted the use of "nt sequence

similarity" from the revised manuscript.

2. The aligner used (ClustalW) may not be the most appropriate for such rapidly evolving viral proteins and should be changed. The alignments (nucleotide or amino-acids) may be made available.

Our reply

According to the reviewer's comment, we re-aligned the sequences and re-constructed the ML tree. We replaced the **Fig 1A** with the new version.

3. Bootstraps superior to 50% (highlighted in the figures) should not be considered as significant branch support. The common cutoff in the literature is above 75-80%.

Our reply

In the revised manuscript, the cutoffs of bootstrap values are >80% (*) and >95% (**) (page 32, line 737).

4. The "phylogenetic tree" presented in figure 4C should be taken out entirely: 12 nucleotides are not informative enough for a phylogeny. This analysis resembles more a cluster analysis.

Our reply

According to the reviewer's suggestion, we omitted this figure from the revised manuscript.

5. On a related note: Is the DRMR motif in Vif also found in monkey SIVs?

Our reply

Referring to previous reports, Old World monkey (OWM) SIV Vif proteins can degrade the A3F proteins of their hosts. For example, SIVmac 239 Vif degrades rhesus macaque A3F (Hultquist et al, J Virol, 2011. PMID 21835787). However,

it remains unclear which domain(s) in SIV Vif are crucial to degrade OWM A3F. To address the reviewer's concern, we analyzed the amino acid/nucleotide sequences of OWM SIV Vifs. We noticed that the Vif sequences of OWM SIVs were quite different from those of HIV-1M; for instance., the nucleotide similarity between HIV-1 NL4-3 (accession no. M19921.2) and SIVmac 239 (accession no. M33262.1) is only 21.5%. Also, the Vif sequences were very diversified among SIV strains (i.e., different host species). Moreover, we could not detect the DRMR motif in any OWM SIV Vif proteins and these sequences are too diversified to align together with the Vif sequences of HIV-1M and other great ape lentiviruses (eg, SIVcpz and SIVgor). Therefore, we think this would be beyond the scope of our study and did not include these observations in the revised manuscript.

6. In Figure 4, the authors discuss the SIVgor Vif ability to degrade gorilla A3F in a DRMR-independent manner, but not the fact that SIVcpz Vif showed the same ability (Fig 4E). This should be added to the text and the conclusions/model brought by the authors should be modified accordingly. Experiments testing the same Vif strains/mutants against human A3F and chimpanzee A3F would be an important addition.

Our reply

Regarding *"the authors discuss the SIVgor Vif ability to degrade gorilla A3F in a DRMR-independent manner, but not the fact that SIVcpz Vif showed the same ability (Fig 4E). This should be added to the text and the conclusions/model brought by the authors should be modified accordingly"*; we appreciate the reviewer for the important suggestion. According to the reviewer's point, we modified the text of revised manuscript (page 14, line 324).

To satisfy the latter part of the comment, according to the reviewer's suggestion, we performed an additional experiment using human A3F. The result is shown in **Fig 4G** of revised manuscript. We think this is a good control for the result of gorilla A3F (**Fig 4F** of revised manuscript) in terms of the importance of DRMR motif (F1 box). We would like to thank the reviewer again for the important suggestion.

Regarding cross-species lentiviral transmission events in great apes; as shown in **Fig 1B** of the revised manuscript, there are three patterns, (1) from chimp to gorilla (focus of this study), (2) from chimp to human, and (3) from gorilla

to human. However, there are no cases of “from human/gorilla to chimp”. Therefore, we thought the experiment using chimp A3F is not essential in this study.

Minor comments:

In Figure 3, although most SIVgor Vif have an E at the 16th position discussed as of primary importance for gorilla A3G degradation, one of the SIVgor Vif sequences (SIVgorBQID2) presents a Q at this position (panel A). Considering that the SIVcpz M16Q mutant in panel C does not degrade gA3G, have the authors tested the capacity of the SIVgorBQID2 strain to degrade gA3G? If not in the capacity to perform additional experiments, the authors should discuss this point in the manuscript.

Our reply

Please see the *vif* sequence of SIVgor strain BQID2 (accession no. KP004991):
atggaaaacaaatggcaggt**R**ctgatagtatggcaag**W**ggacagacagaaggtgaaagcatggaaca
gcctggtgaaataccataagtataggtccaaaaaggccaaagattggttttacagacaccattatgaagccac
aaatccaagagtcagttcaggtgtatatattccagtaggtggtgcttgcataatagtgaccacatattggggtttg
atgcca**R**gggaag**M**agctga**R**caattgggaaatggggttagtatagaatggcagctaaggaattatagaa
cacagattgatcctgaaacagcagacaggatgatacacctgtattat**Y**tttc**M**tgttttacagagtcagcaatc
aggaaagccatcttagggcaaaaggtgttacccacttgtgaataccctgcaggacatagtcaggtagggacc
cttcaattcctagctttgagggtattagtaaaaacaagaaaatttagacctcccttacccagtgtccagaagttag
cagaagatagatggaacaggtacccgaggatcagggaccagctagagagccattcaatgaatgggcactg
gaagttctggaagaactga

As shown above, 7 out of the 597 ORF nucleotides were ambiguous (e.g., R, W, M or Y), and therefore, 4 out of the 7 codons containing these ambiguous nucleotides were unable to be defined as the codon encoding single amino acid (for instance, there are 2 “X”s in the amino acid sequence of BQID2 in Fig. 2I of the revised manuscript [Fig. 3A of the previous version]. We noted that “X is an undefined amino acid because of nucleotide ambiguity” in the legend of revised manuscript [page 34, line 783]).

We also found that the full-length sequence of SIVgor strain BQID2 (9,241 nucleotides; accession no. KP004991) contained 53 ambiguous nucleotides.

These observations suggest that the sequence quality of this strain is not so good, and therefore, a Q at position 16 of BQID2 Vif may also due to the low quality of sequence. The sequence ambiguity may affect the experimental result, and it was difficult for us to choose an “authentic” BQID2 sequence from the public database for artificial DNA synthesis and subsequent virological experiments. Therefore, we could not address this issue. We explained this possibility in the revised manuscript (page 9, lines 210-215). We think the editor and reviewers for understanding this explanation.

The size of the figures is slightly small and may benefit from being bigger.

Our reply

To satisfy this point, we modified the figures to the best of our abilities. Now the size of characters (ie, point) in the figures are 6 pt, which satisfies the journal’s criteria.

The use of "gorilla peripheral blood" is mentioned in the text but does not appear in the Method or Figures. Can the authors check this point?

Our reply

Thank you very much for finding our mistake. This is not related to this manuscript, and was removed from the revised manuscript.

Reviewer #3 (Significance (Required)):

This study is very well conducted and the experiments are clearly described. Given the difficulty of constructing vif chimeras and mutants, the experiments related to them are particularly interesting.

This study will be of interest as a contribution to the complete description of the Vif-APOBEC3G interface. This interface has been of particular interest and extensively studied due to the important role of A3G in preventing HIV infection when not counteracted by the virus.

Furthermore, this study brings important findings on the origin of HIV-1 in humans, as the origin of SIVgor represents the ancient origin of HIV-1 groups O and P.

Our reply

We would like to appreciate the reviewer for the positive comments such as *"This study is very well conducted and the experiments are clearly described"* and *"Given the difficulty of constructing vif chimeras and mutants, the experiments related to them are particularly interesting"*. Particularly, we are very happy to hear the reviewer has recognized the difficulty of constructing vif chimeras. To complete our study, we addressed all the concerns raised by the reviewer. We hope the revised manuscript is now acceptable in the journal.

Reviewer's field of expertise:

phylogenetics, virus-host interactions, HIV, restriction factors.

Of note: The analyses and results on the structure homology models (Fig 3D-F) appear well conducted and convincing and they are a very interesting addition to the study. However, they are outside of my direct field of expertise and thus it is difficult to confirm their validity.

REFEREES CROSS COMMENTING

I agree with all the comments that were raised by the other reviewers. They are fair and balanced.
